# Supplementary material for: A neuron model with unbalanced synaptic weights explains the asymmetric effects of anaesthesia on the auditory cortex
Source: PLoS Biol. 2023 Feb 21;21(2):e3002013. doi: 10.1371/journal.pbio.3002013 (PMC10013928; doi:10.1371/journal.pbio.3002013)
Supplement: Attachment — Submitted filename: Response_to_Reviewers.docx [file pbio.3002013.s010.docx]

*Fachbereich Biowissenschaften
Max-von-Laue-Str.13,
Biologicum, Gebäude A
60438 Frankfurt am Main*
[*www.bio.uni-frankfurt.de*](http://www.bio.uni-frankfurt.de)

Dear Editors,

We would like to thank you one again for giving us the opportunity to submit a revised version of our manuscript to *PLOS Biology*. In addition, we would like to thank the reviewers for their insightful comments and suggestions. As you will notice, while preparing this revision we responded to all the queries made by the reviewers. The result is a manuscript that has considerably improved compared to our initial submission.

In summary, the new version incorporates:

- 2 more main figures, in which we tested two alternative models requested by Reviewer #2.
- 2 more supplementary figures supporting one of the two new alternative models.
- Replacement of Fig 1 in order to better introduce the goal of our study, as suggested by Reviewer #1.
- Organizational changes in the Discussion in order to improve the reading.

Below, we will address point-by-point all the comments from the 3 reviewers.

We look forward to hearing from you,

Luciana López Jury

Julio Hechavarria

--

REVIEWS:

Reviewer #1: In "A neuronal model with unbalanced synaptic weights explains asymmetric effects of ketamine in the auditory cortex", Lopez-Jury and colleagues use a combination of computational modeling and in vivo electrophysiology to understand the impact of ketamine/xylazine (KX) anaesthesia on auditory cortical neurons responses to various contexts. The two main contexts that are examined are an echolocation/navigation context and a social (distress call) context. The authors varied several parameters in a simplified cortical model - the degree of adaptation of synaptic inputs, the degree of pre-synaptic or the degree of post-synaptic depression induced by KX and examined how varying these parameters affected model responses to contextual stimuli. They then used hypotheses generated by these data to design in vivo experiments to test these hypotheses, including several experiments involving testing the same neurons pre- vs. during- KX anesthesia. They also designed novel
experiments using chimeric stimuli to separate temporal from frequency effects, and ultimately concluded that KX effects are dominated by its impact on frequency.

In general, I think this manuscript represents an excellent example of cross-fertilization of computational modeling with electrophysiology experiments. It is not clear to me that the results will have a general impact on our understanding of neural processing of context, or simply in how KX affects neural networks.

Thank you for this insightful comment. Indeed, the main question of this study is: *how KX affects neural processing of context*. We believe that this is an important question as many past (and still ongoing) studies rely on anesthetized preparations. What we show is that the effects of ketamine are not easy to “regress out” of the neural signal, and this is particularly so when studying the effects of acoustic context on neural responses. Since context dependence of neural processing is a growing field, we think our study could offer valuable insights for planning future experiments and for interpreting results obtained under ketamine.

The study is complicated and I had a hard time following the manuscript. I think it may help to make some small organizational changes in the manuscript. For example, it seems odd to begin the paper with PubMed data on the importance of KX. These types of data are typically found in grant proposals or lectures, not papers.

Thank you for your comment and we apologize for the complicated structure. You are, of course, correct. We have removed the figure with the PubMed data from the beginning of the paper and we have replaced it with a figure that hopefully motivates and clearly states the goal and the impact of the current study. New Fig 1 can be found below.

In addition to changing Fig 1, we have done organizational changes in the Discussion section too. We think this modification indeed improve the reading experience. For example, now the subsections of the Discussion have clearer and more concise titles:

- *Anaesthesia unbalances inputs in multifunctional neurons in the auditory cortex*
- *Effects of ketamine on neuronal responses*
  - *The activity of high-frequency tuned inputs to pyramidal neurons is selectively reduced*
  - *Anaesthesia increases non-specific adaptation of cortical neurons*
  - *Excitatory synaptic receptors are affected asymmetrically by anaesthesia*
- *Effects of anaesthesia on interneurons and their role in context-dependent processing*

I think it'd be better to have a figure 1 that really motivates the paper. What phenomenon are the authors attempting to understand? Clearly it is the effect of context on acoustic responses.

Thanks for this suggestion. The phenomenon we attempt to understand is the effect of the acoustic context on acoustic responses. However, the particular question is how does KX affect such phenomenon. Following your suggestion, we decided to start the manuscript with a figure in which we first show the behavioural relevance of the phenomenon (Fig 1A). Then we illustrate a representative and clear example of the phenomenon itself (B) and, finally, we set the lines on how we intend to answer our question (C). Please see new Fig 1 below.

**Fig 1. Effects of anaesthesia on context-dependent processing of vocalizations in the cortex of bats.**

(A) A representative spectrogram of natural bat vocalizations obtained from a group of bats. The audio recording is composed of a mixture of echolocation pulses and social calls uttered by several conspecifics roosting together. The colour bar indicates sound amplitude (arbitrary units). (B) Response of one cortical neuron to playbacks of echolocation and communication calls heard in silence (left) and after acoustic contexts (right). The responses were recorded in the awake state. Data from (Lopez-Jury *et al.*, 2021). (C) Top left: Location of the bat auditory cortex (AC). The HF cortical fields that contain multifunctional neurons (such as that represented in B) are highlighted in red. Below, a characteristic multipeaked frequency tuning curve (FTC) from these neurons is shown. Right, an integrate-and-fire neuron model reproduces the context-dependent processing observed experimentally in awake bats. In theory, the known effects of ketamine on evoked responses can be used to predict ketamine effects on context-dependent processing in the bat cortex.

I think providing the reader with a clearer example of the physiological phenomenon will help us to understand why the subsequent manipulations were made.

Thank you for this comment. In the new Fig 1B, we have included a clear example of the physiological phenomenon. We agree that this will help understand the phenomenon. In the next panel (Fig 1C), after the phenomenon is presented, we illustrate the model and model nodes that we will manipulate to predict the effects of KX on the phenomenon described above. The question marks in the diagram of the neuron model indicate the possible nodes that can affected by KX and that could thus influence cortical processing of acoustic contexts.

The rest of my comments are minor:

1.        "Navigation" and "echolocation" appear to be used interchangeably. Would suggest using just one.

True. Sorry about that. Now we have replaced all the instances where we used “navigation” to “echolocation”. All replacements are highlighted in the new manuscript.

2.        The phrase "associated to" is used often. It should be "associated with"

Thank you. We corrected this typo throughout the manuscript.

3.        Line 179, place a comma after "regions"

Thanks. We removed this line because we included a clearer way to summarize the results of that section. You will find the new paragraph highlighted in the new version of the manuscript.

4.        Line 258, should be "evoked"

Done! Thanks.

5.        Line 532, please provide actual dose (in mg or mg/kg) of ket and xylazine here

Thank you. This is actually very important to clarify. The actual dose is now included in the manuscript, and it is 7.5 mg/Kg of ketamine and 16.5 mg/Kg of xylazine.

6.        Fig 5A legend - spelling "anestheitzed"

We could not find the misspelling "anestheitzed".

7.        In general for the figures, whenever rasters are shown, it would be helpful to have a diagram of the stimulus shown above or below the raster

We agree. In the new Fig 3B, an oscillogram of the stimulus was added on top of the respective three simulated rasters. Also in the new Fig 6D-E, the respective oscillograms of the stimuli were added on top of the awake and anaesthetized *in vivo* data rasters. In the same way, new Fig S6 needed the respective stimulus too, and it was added. The rest of the rasters presented along the manuscript already had a diagram of the stimulus.

8.        Also, in general for the figures, it would be useful if somewhere on the figure it is stated if the data are real neuronal data or model data

We agree that the combination of experimental and modelling data could be confusing throughout the figures. In the new version of the manuscript, the figures 3, 5, 6, 9, 10, S6, S7 and S8 include a label either “*in vivo* data” or “model data”, accordingly.

Reviewer #2 (Jordan David Chambers): In this manuscript, the authors investigated how the auditory cortex responds to vocalizations under ketamine induced anaesthesia and in wakefulness. They used a combination of electrophysiological recordings and mathematical modelling. The electrophysiological recordings showed ketamine effects on context-dependent processing varied with the frequency composition of the sounds used as context. That is, ketamine effected communication (low frequency) context and not echolocation (high frequency) context. The mathematical modelling explored parameters relating to cortical input firing rate, presynaptic adaptation, and postsynaptic adaptation. The model only reproduced all electrophysiological recordings when ketamine affected only high-frequency cortical inputs and both presynaptic and postsynaptic adaptation.

Overall, the manuscript is well written. The methodology is sufficient to reproduce the experiments. The electrophysiological data and analysis fully support the author's claims. The computational model presented supports the author's claims. However, the computational model is relatively simple, which may or may not be misleading. The findings presented are novel and will be of interest to researchers in the field of auditory processing and more generally to sensory processing. The findings relating to ketamine and anaesthesia will be of interest to all neurosciences.

Major concerns:
1.        Ketamine mainly affects glutamatergic transmission inhibiting NMDA receptors, but the computational model does not contain inhibitory neurons. The authors recognise this and discuss the limitations of their model but trying to model the affects of ketamine without inhibitory neurons will significantly reduce the impact of the computational model.

Thank you for your suggestion, we believe that the implementation of the new models really improved our study and it strengthens the results. In this version of the manuscript, we have added two alternative models that include inhibitory neurons in a cortical circuit model. Below you can see diagrams of each of the alternative models developed:

These models and their predictions have been added in the Results section, particularly in the initial part of *naïve* models. The conclusions that we reached by modelling interneurons were: (1) these two alternative models behave similar to each other in terms of context-dependent processing: (2) none of them predicts a significant effect of the anaesthesia on the phenomenon studied (stimulus-specific suppression). (3) Both models may predict an enhancement of the cortical responses to sounds if the ketamine affects selectively the excitatory inputs that impinge onto the inhibitory interneurons, this is due to disinhibition of the pyramidal neurons.

Considering that none of these predictions were confirmed in our experimental data, we discarded the possibility that the effects driven by KX in context-dependent processing was due to the antagonist action of ketamine on interneurons. We clarified in the Discussion that this does not imply that inhibition is not playing a role in the processing of context in acoustic responses. Considering that disinhibition of the cortex caused by ketamine has been extensively discussed in the literature, we briefly discuss possible explanations of our results.

The new manuscript includes these results as an additional section in the Results called: *“Predicted effects of anaesthesia in a cortical circuit that includes inhibition (naïve modelling)”,* as well as a new section in the Discussion called: *“Effects of anaesthesia on interneurons and their role in context-dependent processing”*.

2.        The mathematical model explores parameter spaces relating to presynaptic and postsynaptic adaptation. This adaptation is modelled by increasing the threshold, which then decays with a time constant. This sort of adaptation can easily be applied to any ion channels in the model, but this model only contains a single excitatory conductance (in addition to a leak conductance and a stochastic conductance). By including more conductances and exploring the adaptation for each conductance, the model could easily provide an explanation as to why ketamine is affecting high frequency presynaptic adaption and not low frequency presynaptic adaption (e.g. ketamine is affecting cells that contain a particular ion channel). At the moment, the computational model requires a different affect on low frequency input and presynaptic adaptation compared to the high frequency input and presynaptic adaptation. This is a not a model providing insights into how ketamine has different affects on
communication context compared to echolocation context, but simply a model that separates the low and high frequencies to replicate the experimental observations. The authors do discuss the evidence for a peripheral affect in the cochlear, but if this is the only mechanism, then the findings are of less importance to the sensory processing and general neuroscience.

We are very grateful to this comment as it led us to propose an updated model that is more biologically and better at explaining frequency-specific synaptic effects of anaesthesia. Following your suggestion, we added a second excitatory conductance to the previous model and we assumed that each of these two excitatory conductances were associated with a different synaptic receptor in the pyramidal neurons (called R_1_ and R_2_ for simplicity). In addition, we assumed that ketamine would affect selectively one of these two synaptic receptors (in this case, R_1_). A diagram of the new model is depicted below:

From the new simulations, we showed that these two receptors must be associated with different inputs’ tuning in order to reproduce the experimental results. This broadens the impact of our modelling experiment because it does not only predict the effects of ketamine in the framework of context-dependent processing, but also it provides a prediction about the general physiology of pyramidal neurons. The excitatory transmission in the cortex is glutamatergic and that it can co-activates NMDA and AMPA receptors. We discuss the possibility that R_1_ and R_2_ receptors in the new model may represent indeed different ratios of AMPA and NMDA receptors in the pyramidal neurons.

What we observe in our updated model is that the differences in terms of the dynamics of synaptic conductance in both receptors is rather modest (new Fig 11B right panel). The temporal course of the conductance across the receptors only differ when synaptic depression is noticeable. Different ratios of AMPA and NMDA receptors could exhibit more modest changes in conductances than two completely different synaptic receptors. However, we cannot rule out the possibility that these represent two actual different synaptic receptors with similar dynamics.

Secondly, it is known that ketamine affects NMDA, but not AMPA receptors. Therefore, it makes sense to propose a model in which one channel is more affected than the other. In this case, ketamine acts as specific NMDA blocker and it can give us some hints about the receptors underlying the processing of sounds.

The new manuscript includes these results as an additional section in the last part of the Results called: *“Two excitatory conductances in pyramidal neurons better explain experimental data”,* as well as a new section in the Discussion called: *“Excitatory synaptic receptors are affected asymmetrically by anaesthesia”*.

Minor comments:
1.        Line 266-267 The sentence "The same gap was used in the stimuli used in…" is confusing.

Thank you, now it reads: “The same gap was used to stimulate the neurons in the awake preparation.”

2.        Line 272: "Despite of" should just be despite?

Yes, fixed it.

3.        Throughout the text adaption and adaptation are used. While both are acceptable, it is probably best to just use adaptation throughout or at least be consistent

Agree. But we couldn’t find any instances of the word “adaption”.

4.        Line 435 "both central and peripheral" but there is no discuss or evidence suggested for the central sources. Please discuss how a frequency-specific decline could occur in the central auditory system.

That is true, thanks for pointing it out. In the new version of the Discussion, we have added a paragraph that says: “… because ketamine selectively blocks NMDA receptors in central neurons, a variation of the effects of these receptors across the tonotopy could explain a frequency-specific effect. Such variations have been shown in terms of neuron excitability (Brew & Forsythe, 2005), amount of receptors (Yang *et al.*, 2009) and synaptic properties (Oline & Burger, 2014)”.

5.        Line 495-497: It is not clear how different patterns of synaptic activation may explain the contradictory effects of ketamine.

Yes, in fact this paragraph is now deleted. We agree that the sentence was a bit vague, so we decided to mention more concrete reasons of why there could be contradictory effects of ketamine across several studies. Now the paragraph reads like this: “Contradictory effects of anaesthesia in the processing of sounds can be explained by differences in (i) the methods used to measure neural activity: population activity versus single neuron spiking, (ii) the area and the type of neurons recorded, (iii) the animal model and (iv) the sound used to stimulate the neurons: i.e. artificial versus natural sounds.”

Just to clarify, the previous idea of “different patterns of synaptic activation” was thought in the sense that the stimulation with a pure tone activates different synapses in the dendritic tree of a neuron compared to the stimulation with a natural sound. These differences include different type of synapses (GABAergic, glutamatergic, etc), different amount of synapses (probability of opening of channels), different locations of the active synapses and different timing of activation. All of these possible scenarios could explain variability in the outcome of blocking NMDA receptors with ketamine.

6.        Line 695: It is not clear where the variability in the model arises from to require 20 trials each. Is it the Poisson process for the inputs? The stochastic conductance?

The variability arises from the Poisson process used to create the spike trains of the inputs together with the stochastic conductance, added in the neuron model as a Ornstein-Uhlenbeck process. In the new version of the Methods, we wrote down the equation that describes the membrane potential of the neuron model. There, the stochastic conductance is explained in more detail.

The reason of 20 trials is to quantify the output of the model as similar as possible to the output of the experimental data. In all the neurons that we recorded in awake and anaesthetized bats, we performed 20 repetitions of the same stimulus (20 trials). It is worth mentioning that 50 neuron models with 20 trials each is, in theory, the same as 1000 repetitions of the same simulations. In our previous work (Lopez-Jury *et al.*, 2021), the parameters associated to neuronal noise were set in order to reproduce the real behaviour of the neurons. In the model, the variability across trials resembles the variability across trials observed *in vivo*.


Reviewer #3: The authors explore a very important question in neurophysiology and their study sheds light on the complexities of the effect of anesthesia for electrophysiological recordings. The manuscript is clear and well written. The authors present sound data that supports their conclusions regarding the effect of Ketamine on high frequency tuned neurons and how this may affect the auditory processing of context dependent natural sounds.
Their implementation of the model using the known effects of ketamine and the differences that arise when comparing it to the real data provides convincing evidence that there are other effects to Ketamine that may not be widely understood. Here, for their set of experiments, adding the effect on high frequency components provides a model that reliably fits the data. Nevertheless, it opens the question of what other effects may not be evidenced because of the experimental paradigm. This work moves the field forward by demonstrating this effect and providing the groundwork for this to be tested against other experimental paradigms that may bring forward other unknown effects of anesthesia in neurophysiology.

I have only minor comments:

Line 80: suggested change of words "enable" instead of "allow"

Done. Sounds much better. Thanks!

Figure 6: the red and orange are very similar and the dark blue and lighter blue are very similar too. They are hard to see and I recommend the authors use colors that are more discriminable. Although clear in figure 6c, these small color differences are not explained in the figure legend, the authors should add this.

Thanks for noticing this issue. Indeed, the colours were not so different to each other. This is now corrected by using only two colours that allow to easily discriminate between awake and anesthetized data. Also, we added the labels “match” and “mismatch” that were missing in the panel D and E.

Line 486: The start of the sentence with the word "Besides" is confusing. I would advise the authors to reword.

We reworded as follows: “Although anaesthesia showed asymmetrical effects on the stimulus-evoked activity in our recordings, the effects were solely suppressive…”

**References**

Brew, H.M. & Forsythe, I.D. (2005) Systematic variation of potassium current amplitudes across the tonotopic axis of the rat medial nucleus of the trapezoid body. *Hear Res*, **206**, 116-132.

Lopez-Jury, L., Garcia-Rosales, F., Gonzalez-Palomares, E., Kossl, M. & Hechavarria, J.C. (2021) Acoustic Context Modulates Natural Sound Discrimination in Auditory Cortex through Frequency-Specific Adaptation. *J Neurosci*, **41**, 10261-10277.

Oline, S.N. & Burger, R.M. (2014) Short-term synaptic depression is topographically distributed in the cochlear nucleus of the chicken. *Journal of Neuroscience*, **34**, 1314-1324.

Yang, S.M., Toshihiko, K., Shu–Ping, C., Wen–Ming, W., Ayumi, M., Mikiya, A. & Toshio, Y. (2009) Optical recordings reveal the differential distribution of glutamatergic transmission in the cochlear and vestibular nuclei in the newborn mouse brainstem. *Journal of Otology*, **4**, 106-114.
